# Persistent Deficits after an Achilles Tendon Rupture: A Narrative Review

**DOI:** 10.1155/2022/7445398

**Published:** 2022-07-16

**Authors:** Rikke Hoeffner, Rene B. Svensson, Nicolai Bjerregaard, Michael Kjær, Stig Peter Magnusson

**Affiliations:** ^1^Institute of Sports Medicine Copenhagen, Department of Orthopedic Surgery, Copenhagen University Hospital—Bispebjerg and Frederiksberg, Copenhagen, Denmark; ^2^Center for Healthy Aging, Department of Clinical Medicine, University of Copenhagen, Copenhagen, Denmark; ^3^Department of Physical and Occupational Therapy, Copenhagen University Hospital—Bispebjerg and Frederiksberg, Copenhagen, Denmark

## Abstract

Persistent muscle weakness, tendon elongation, and incomplete return to preinjury level are frequent sequelae after acute Achilles tendon rupture, and evidence-based knowledge of how to best rehabilitate the injury is largely absent in the literature. The objective of this review is to illuminate and discuss to what extent an Achilles tendon rupture affects muscle, tendon, and function when assessed with the Achilles tendon total rupture score (ATRS), muscle strength, muscle cross-sectional area, tendon length, and the heel-rise test. The patient-reported outcome measures (PROM) data in the literature suggest that the recovery takes longer than 6 months (ATRS, 70 out of 100), that one-year postinjury, the ATRS only reaches 82, and that this does not appear to noticeably improve thereafter. Loss of muscle mass, strength, and function can in some cases be permanent. Over the first 6 months postinjury, the tendon undergoes elongation, which appears to be negatively correlated to heel-rise function. More recently, there has been some interest in how muscle length and excursion is related to the reduced function. The available literature indicates that further research is highly warranted and that efforts to restore normal tendon length may improve the likelihood of returning to preinjury level after an Achilles tendon rupture.

## 1. Introduction

Leisure time physical activity is unquestionably associated with numerous health benefits; however, these recreational activities are also accompanied with some increased risks of injury. For example, running, jumping, and agility activities that involve eccentric loading and explosive plyometric contractions can be associated with an increased risk of Achilles tendon rupture, which is a relatively frequent injury in high-impact sports, including badminton, volleyball, and soccer [1–5]. Acute Achilles tendon ruptures occur in both men and women, but is most frequent in men of 30–50 years of age that participate in recreational sports periodically [5–7].

The incidence reaches 31/100 000/year and has been reported to be on the rise [5–9]. Despite the fact that a rather sizeable number of people suffer this injury, evidenced based knowledge of how to best rehabilitate following a rupture remains unclear, and this lack of knowledge likely contributes to the reports of persistent muscle weakness, tendon elongation, and incomplete return to recreational preinjury level [10–25], and for high level athletes, a rupture of the Achilles tendon can be career ending [20, 26]. In this review, we will focus on effects of Achilles tendon rupture on tendon and muscle structure and function without distinguishing between conservative and surgical treatment approach, since similar effects are observed with both forms of initial injury management [14, 22, 25].

## 2. Patient-Reported Outcome Measures

**Figure 1:**
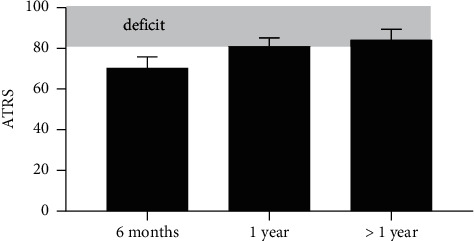
The mean (and corresponding 95% CI across study) ATRS of pooled data published six months, one year, and >1 year after Achilles' tendon rupture. The shaded bar indicates the average deficit 18% after 1 year. These data represent a total of 3574 subjects from the following references [11, 14, 15, 24, 32–65].

Many outcome measures can be used to evaluate the effect of an intervention, but patient-reported outcome measures (PROMs) that emphasize how the patient experiences the impact of the disorder or injury have become the gold standard in clinical research [27]. When evaluating patients with a more specific diagnosis such as Achilles tendon rupture, a condition-specific PROM is most appropriate [28]. Several PROMs have been used for Achilles tendon ruptures, such as the VISA-A [29] and the foot and ankle outcome score (FAOS) [30], but the most widely used is the Achilles tendon total rupture score (ATRS), which has been validated as a condition-specific PROM [31]. The ATRS questionnaire consists of 10 questions that reflect symptoms and physical activities, and it is answered using an 11-grade Likert scale. The scale ranges from 0 to 10 with 0 representing major limitations/symptoms and 10 representing no limitations/symptoms. The score of uninjured healthy persons approaches 100, which is the maximal score on the ATRS [31]. Patients report mean scores of 31–56 at three months [10, 11, 13] and 54–87 at six months [11, 32–44]. One year after the rupture, the mean score has been reported to be 74–91 [11, 24, 32, 33, 35, 36, 38–40, 42–55], and it does not appear to appreciably improve further thereafter [14, 15, 24, 39, 42, 46, 47, 56–65].  Figure 1 shows the average ATRS score based on the aforementioned studies at different timepoints.

Collectively, these PROM data suggest that the recovery takes longer than 6 months (ATRS 70) and that patients do not necessarily recover completely. In fact, one-year postinjury, the ATRS only reaches 82, and beyond one year, there does not appear to be a numerically substantial improvement in the average ATRS reported data. Since it has been shown that ruptured Achilles tendons do not appear to regain normal metabolism, blood flow, and stiffness until about 12 months postinjury [11,66], the usefulness of ATRS in the early stages, e.g., at three months, of the recovery process is questionable.

## 3. Muscle Strength and Cross-Sectional Area

Attempts to understand the lack of a complete recovery to preinjury level has mostly focused on muscle strength and mass. It is commonly accepted that a lack of complete recovery following Achilles tendon rupture is largely related to a lack of muscle strength in the medial gastrocnemius and soleus muscles, while the mean volume of the flexor hallucis longus muscle has been reported to be greater on the affected side, which may reflect a compensatory hypertrophy [67]. This notion is based on the fact that muscle weakness can persist after surgery [12–23] and may even be present a decade after the injury [23]. There is a large variation in the methodology by which muscle strength has been assessed (e.g., isometric/eccentric/concentric, various speeds, and knee extension/knee flexion); however, plantar flexion strength deficit on the injured side has been shown to be up to 49% [68, 69] of the uninjured side at three months, which is perhaps not surprising since the injury will require some period of immobilization irrespective of surgery or conservative treatment and, thereafter, a rather gradual progression of loading rehabilitation. However, it is noteworthy that after one to several years after the injury, there is still a considerable side-to-side deficit that can be as high as 10–35% [19,68–75], which strongly suggests that the loss of muscle function is long-lasting.

Because muscle strength does not always recover fully after an Achilles tendon rupture, the extent to which muscle mass can recover following the injury has also received attention. This has most commonly been assessed by measuring the anatomical cross-sectional area (ACSA) using magnetic resonance imaging or ultrasound since calculating physiological cross-sectional area (PCSA = muscle volume/fiber length), which more accurately reflects the force-generating capacity of the muscle [76], is challenging clinically. Given the inherent immobilization and relative inactivity associated with an Achilles tendon rupture, it would be expected that some atrophy occurs in the initial months [77]. In healthy uninjured adults, three months of bed rest is associated with a substantial reduction (28%) in CSA of both the medial gastrocnemius and soleus muscle; however, this loss of muscle mass will be completely recovered in the subsequent three months even without any specific countermeasures [78]. Similarly, muscle function appears to fully recover within 6 months after 90 days of bedrest [79]. Because of the remarkable muscle plasticity in response to inactivity/activity, one would also anticipate that any loss of muscle mass (and function) associated with the initial weeks of immobilization and subsequent months of reduced activity would be recovered 12 months after an Achilles tendon rupture. However, several studies report a sizeable and apparently persistent loss of muscle CSA of the triceps surae muscle group. Studies have found a 9–25% side-to-side difference in muscle CSA at 12 months [22, 80] and a 11–15% difference 3–13 years after the injury [67, 81], which collectively suggest that the loss of strength and mass may be permanent in some cases, in contrast to what would be expected from immobilization alone.

This aforementioned loss of both muscle mass and strength has prompted researchers to focus on early mobilization following repair of the ruptured tendon with immediate full weight bearing [75, 82, 83] in an attempt to minimize atrophy. However, these efforts have not been proven to effectively counteract the loss of muscle mass and function. The well documented muscle plasticity underscores that the loss of muscle mass and strength is likely chronic if not recovered after 12 months and that focus on an accelerated approach after injury is perhaps theoretically not necessary to subsequently recover muscle mass and strength.

### 3.1. Tendon Length

In addition to muscle strength and mass, it is possible that the length of the tendon contributes to the functional deficit. Elongation of the human Achilles tendon during the rehabilitation phase postrupture has not always been the primary focus, but has gained more attention. Various methods have been used to measure tendon length, including ultrasonography, magnetic resonance imaging, and radiography with metal markers, and each has inherent strengths and weaknesses, which also precludes direct comparison between studies. The very first report was published almost four decades ago [84]. It demonstrated that there was separation (2-3 mm) of the sutured ends already in the initial four days after the surgical repair despite immobilization and that separation seemed to continue for about 1.5 months. Two decades later, a similar report was published that showed a separation of 5–9 mm in the initial 6 weeks and up to 11 mm at 12 weeks [85]. Since then, several studies have shown that this elongation can reach values of 10–20 mm [11,13,14,22,45,84–88], that the progression appears to be prominent in the initial months after the injury, and that it may not stabilize until around 6 months [11]. In fact, it has been shown that only 50% of the total elongation takes place in the initial three months after surgery and the remaining 50% in the subsequent three months [11]. The magnitude of lengthening can be quite substantial and often correspond to approximately 10–20% of the entire length of the free tendon and in some cases, as much as 50%, which impacts muscle function dramatically. Importantly, it appears that various rehabilitation regimes postinjury cannot demonstrate a significant reduction of the elongation [11]. Aside from the dissimilar methodology used to assess tendon length, it should be noted that some data are based on measurement of the tendon associated with the gastrocnemius medialis muscle and some with the soleus muscles (i.e., “free tendon”), but rarely both with one exception [89]. The free Achilles tendon is considered a single homogeneous structure, but it consists of distinct portions from each of the three muscle compartments of the triceps surae [90], and it may be relevant to examine the different tendon portions.

The elongation over several months prompts questions surrounding the healing process of tendon tissue after rupture and how load progression should be timed, which are questions that remain largely unanswered. Tendon tissue is metabolically active and responsive to loading [66,91], and using positron emission tomography, it has been shown that compared to intact Achilles tendons, the metabolic demand in ruptured tendons was higher during walking by 6, 3, and 1.6 fold at 3, 6, and 12 months following repair, respectively [66]. Moreover, glucose uptake was negatively correlated to ATRS six months after the repair, and tendon blood flow seemed to normalize between 6 and 12 months [66]. It has also been shown that the stiffness of the tendon increases up to 12 months postsurgery [11,87]. Collectively, data on tendon elongation, stiffness, metabolism, and blood flow suggest that the healing process likely takes up to a year and maybe even longer.

### 3.2. Functional Outcomes

In addition to quantifying the patient-reported outcome, it is informative to also assess a functional outcome following an Achilles tendon rupture. The most commonly used functional test is the heel-rise test, which is a valid and reliable method of evaluating muscle endurance (rather than strength per se) [12,92]. It is performed standing on one foot on a 10° incline board with the knee kept straight, while as many heel-rises as possible are performed to the greatest possible height at a rate of 30 heel-rises per minute. The numbers of heel-rises and the height of each heel-rise are documented, and the total amount of work can be calculated [12,13]. After six months, the number of repetitions (75–84%), heel-rise height (61–69%), and work (44–52%) of the injured leg remains substantially deficient relative to the healthy [11]. This deficit in heel-rise height (72–84%) and work (63–70%) remains even beyond 12 months [10,11,24,89,93]. Muscle mass normally recovers within months after a period of immobilization as discussed above, but the fact that reduced muscle strength and atrophy remain several years postinjury and that the heel-rise function can also remain markedly reduced years after the initial injury suggest that it may be a permanent loss of function.

The tendon length after rupture appears to influence the heel-rise height. It has been shown that there is a negative correlation between deficit in heel-rise height and Achilles tendon length at six and 12 months [13,46]. A similar correlation has also been shown in persons who experience a functional deficit 2 years after an Achilles tendon rupture, albeit only for the gastrocnemius tendon elongation, but not soleus tendon elongation [89]. These findings suggest that restoring normal tendon length is likely important to achieve optimal function.

### 3.3. Muscle-Tendon Unit Excursion

The reason for incomplete recovery on the heel-rise test remains largely unknown. The triceps surae with its three muscles (gastrocnemii and soleus) and Achilles tendon operate as a functional unit, and although there has been some attention directed to muscle CSA/strength historically and tendon length more recently, little or no consideration has been given to muscle length. The amount of parallel contractile tissue (PCSA), which to some extent is related to the more commonly estimated CSA using magnetic resonance imaging, is associated to the force-generating capacity of the muscle. However, it is also well known that muscle fiber length is proportional to the total excursion of that muscle [94]. As previously mentioned, the Achilles tendon may elongate substantially during the healing period after surgical repair. Therefore, if a concomitant shortening of the muscle takes place, it would reduce muscle excursion and thereby reduce the heel‐rise height [89,95,96]. In fact, persons that have a severely reduced heel-rise height (32%) and elongated gastrocnemius (14%) and soleus (55%) tendons also have a shorter medial gastrocnemius fascicle length (18%) [89]. These data suggest that muscle length and function is impacted by the elongated tendon.

The sarcomere is the functional unit of skeletal muscle: the number of parallel sarcomeres governs the force-generating capacity, and the number of sarcomeres in series governs the muscles total excursion, i.e., joint range of motion [97]. It is well established, albeit mostly based on animal models, that serial sarcomere number can be altered during periods of immobilization in the lengthened or shortened position to optimize the force-generating capacity of the muscle [98, 99]. If the tendon is elongated and the muscle shortened as a consequence, the force-generating capacity could theoretically be maintained if the serial sarcomere number was reduced to maintain the optimal actin-myosin overlap; however, this would also result in reduced muscle excursion. Alternatively, a lack of adaptation in the number of serial sarcomeres would markedly impair both force-generating capacity and muscle excursion. Yet, sarcomere structure and shortening are never measured in the clinical setting, and therefore, these concepts remain to be validated in a human model.

In conclusion, this narrative review highlights the fact that the PROM data presently available in the literature suggest that recovery following total Achilles tendon rupture takes longer than 6 months (ATRS 70), that one year postinjury, the ATRS only reaches 82, and that this remaining deficit does not appear to noticeably improve thereafter. Losses of muscle mass, strength, and function can in some cases be permanent. The tendon undergoes elongation postinjury, and this process can last up to 6 months and appears to be negatively correlated to heel-rise function. There are also some observations that suggest muscle length and excursion is related to the reduce function. It may be that the loss of function relates to the muscle adjustments in response to the lengthening of the tendon. Altogether, the available literature indicates that further research is highly warranted and that efforts to restore normal tendon length may improve the likelihood of returning to preinjury level after an Achilles tendon rupture. [93]
